# Common Traffic Violations of Bus Drivers in Urban China: An Observational Study

**DOI:** 10.1371/journal.pone.0137954

**Published:** 2015-09-15

**Authors:** Qiqi Wang, Wei Zhang, Rendong Yang, Yuanxiu Huang, Lin Zhang, Peishan Ning, Xunjie Cheng, David C. Schwebel, Guoqing Hu, Hongyan Yao

**Affiliations:** 1 Office of Epidemiology, Chinese Center for Disease Control and Prevention, Changping, Beijing, China; 2 Department of Epidemiology and Health Statistics, School of Public Health, Central South University, Changsha, Hunan, China; 3 Changsha Center for Disease Control and Prevention, Changsha, Hunan, China; 4 Department of Psychology, College of Arts and Sciences, University of Alabama at Birmingham, Birmingham, Alabama, United States of America; Beihang University, CHINA

## Abstract

**Objective:**

To report common traffic violations in bus drivers and the factors that influence those violations in urban China.

**Methods:**

We conducted an observational study to record three types of traffic violations among bus drivers in Changsha City, China: illegal stopping at bus stations, violating traffic light signals, and distracted driving. The behaviors of bus drivers on 32 routes (20% of bus routes in the city) were observed. A two-level Poisson regression examined factors that predicted bus driver violations.

**Results:**

The incidence of illegal stopping at bus stations was 20.2%. Illegal stopping was less frequent on weekends, sunny days, and at stations with cameras, with adjusted incidence rate ratios (IRRs) of 0.81, 0.65 and 0.89, respectively. The incidence of violating traffic light signals was 2.2%, and was lower on cloudy than sunny days (adjusted IRR: 0.60). The incidence of distracted driving was 3.3%. The incidence of distracted driving was less common on cloudy days, rainy or snowy days, and foggy/windy/dusty days compared to sunny days, with adjusted IRRs of 0.54, 0.55 and 0.07, respectively.

**Conclusion:**

Traffic violations are common in bus drivers in urban China and they are associated with the date, weather, and presence of traffic cameras at bus station. Further studies are recommended to understand the behavioral mechanisms that may explain bus driver violations and to develop feasible prevention measures.

## Introduction

Bus transportation is among the most common transportation modes in urban China. In 2013, China had a total of 41,738 bus routes that together carried 77 billion passengers and travelled 748,900 kilometers [1]. In Changsha city, where the present research took place, 23% of residents reported riding the bus regularly [2].

Many low- and middle-income nations have witnessed rapid growth of unsafe public transportation as their economies develop. Although such development offers increased economic and social opportunity, it also leads to elevated risk of road traffic injuries [3]. In most countries, buses serve densely populated urban areas and often carry very large numbers of passengers. A single crash can lead to large numbers of injuries and deaths.

The majority of bus crashes are the result of driver negligence and dangerous driving behavior [4, 5]. In one survey from Guangzhou City, China, among all crashes with bus involvement, 42.5% were attributed to bus driver behavior [6]. Others, focused more broadly on all drivers rather than only bus drivers, suggest frequent red-light running rates by road users in Changsha, China [7].

Despite these data, the traffic violation rates of bus drivers have not been reported in China. This study sought to quantify the extent of traffic law violations among bus drivers using observational research strategies.

## Methods

### Ethics statement

This study was considered exempt from requiring informed consent because 1) no study records included private information about bus drivers (name, sex, age, etc.), and 2) it was not feasible to obtain consent of drivers while they were operating buses. The data were not analyzed by or shared with bus companies. This study and procedures was approved by the Medical Ethics Committee of Central South University.

### Study sample

In 2013, there were 135 bus routes run by 13 companies in Changsha city, China. Stratified random sampling was performed to select the study sample. We first divided the 135 bus routes by company (four companies with the smallest bus routes were merged into a single grouping) and then randomly chose 20% of bus routes run by each company to compose the study sample.

### Observational method

The driving behaviors of drivers of each selected bus route were recorded by a single investigator, who rode the bus and discretely recorded the behavior of drivers and relevant influencing factors through the full journey of the bus (from the first stop to the last stop and then back to the first stop). All investigators received substantial training prior to implementation of the observations, and pilot observations of two bus routes were performed prior to data collection to hone the data record form and assure valid data collection.

Each bus route was observed on two different days, one weekday (from Monday to Friday) and one weekend day (Saturday or Sunday). On each observation day, the bus route was observed at four different time periods, including both peak and off-peak hours in the morning, and peak and off-peak hours in the afternoon. According to local regulations [2], peak hours were defined as 07:00–09:00 and 17:30–19:30 on weekdays and 09:00–12:00 and 18:30–20:30 on weekends. Off-peak hours were defined as 09:00–12:00 and 15:00–17:30 on weekdays and 07:00–09:00 and 15:00–18:30 on weekends. During each observation, a full round trip of each bus route was observed, from the first stop to the last stop and then from the last stop to the first stop. In total, therefore, 8 round-trip observations were performed for each selected bus route.

### Road traffic violations of bus drivers

Building from the existing literature [4–6], three types of common traffic violations of bus drivers were observed.

a. Illegal stopping: consisting of stopping outside bus stations, illegal stopping at stations or not stopping at bus stations. According to local regulations [8, 9], ‘stopping outside bus stations’ is defined as ≥3/4 of the bus length is outside the station when the bus stops, or the buses stop at unrecognized places; ‘illegal stopping at stations’ means that a bus stops parallel to other buses that enter the station at the same time, or stops parallel to the bus station with an estimated distance of ≥1.5 meters; and ‘not stopping at the stations’ is defined as not stopping at a station that the regulations require.

b. Violating traffic light signals: According to national regulations [10, 11], violating traffic light signals includes running through red or yellow lights. Running a red light refers to the vehicle entering the zebra crossing and continuing through the intersection while the red light signal is illuminated. Running a yellow light refers to a vehicle that does not stop at the zebra crossing but rather continues running through the intersection while the yellow light is illuminated.

c. Distracted driving: According to the national regulations [11], and building from previous literature [5, 6], we defined distracted driving as instances when a bus driver use his/her mobile phone, ate, smoked, or chatted with passengers while operating the moving bus.

### Influencing factors

Based on the literature and the collective expertise of our research team, we selected the following variables to observe as potential factors that would influence bus driver violations: date (weekday vs. weekend), time period (peak vs. off-peak hours), weather (sunny vs. cloudy vs. rainy/snowy vs. foggy/windy/dusty day) [12–14], cameras at the observation bus stop (yes vs. no) [15], and the presence of an on-duty traffic police officer at the intersection (yes vs. no) [16].

### Statistical analysis

Violation rates and 95% confidence interval (95% CI) were used to quantify the three types of violations, which were calculated as ‘Number of violations/Number of observations*100%’. Considering bus driver violations may cluster within particular bus companies, we used two-level Poisson regression to examine the significance of associations of violations with bus company in the first level and date, time period, weather, cameras at the observation bus stop, and the presence of an on-duty traffic police officer at the intersection as second levels. Adjusted incidence rate ratio (IRR) was used to measure the associations. Stata/IC 12.1 was used to perform statistical analysis. ‘*P*< 0.05’ was considered to be statistically significant.

## Results

### Characteristics of the study sample

In total, we completed 256 round-trip observations on 32 bus routes, recording the bus driver behavior at 7,612 bus stations, 5,656 road intersections, and 14,384 road sections (defined as road segments between intersections and/or bus stops). After excluding rare missing records due to the crowded buses that prohibited valid data collection, we had valid records from 7,611 bus stations, 5,612 road intersections, and 14,277 road sections (Table 1).

**Table 1:** Characteristics of observation of 32 bus routes (Changsha city, 2013).

Bus company	Number of bus routes	Number of observations		
		Road sections	Bus stations	Intersections
No. 1	3	1210	696	448
No. 2	3	1236	660	456
No. 3	2	1196	616	502
No. 4	3	1030	528	384
No. 5	2	1192	644	404
No. 6	2	1276	700	464
No. 7	3	1390	704	600
No. 8	8	3244	1712	1416
No. 9	3	1068	527	424
No. 10^#^	3	1435	824	514
Total	32	14277	7611	5612

^#^: No. 10 bus company was formed by merging four companies with the smallest number of bus routes.

### Illegal stopping

Of 7,611 observations of bus station stops, 1,536 were ‘illegal stopping’, giving an incidence of 20.2% (95% CI:19.3%- 21.1%). These included326 records of stopping outside of bus stations (21% of violations), 456 records of illegal stopping at stations (30%), and 754 records of not stopping at stations (49%) (Table 2). Analysis of contributing factors suggested violation rates of illegal stopping were lower on weekends (adjusted IRR: 0.81) and at stations with cameras (adjusted IRR: 0.89), and were higher on foggy/windy/dusty days (adjusted IRR: 1.53).

**Table 2:** Incidence rate of illegal stopping of drivers at bus stations (Changsha city, 2013).

Independent variables	Number of observations	Incidence rate		Adjusted IRR (95% *CI*)	
		%	(95% *CI*)		
Total	7611	20.2	(19.3, 21.1)		
Week					
Weekdays	3806	22.3	(21.0, 23.7)	1.00	
Weekend	3805	18.0	(16.8, 19.3)	0.81	(0.73, 0.90) *
Time period					
Off-peak hours	3793	21.0	(20.0, 22.3)	1.00	
Peak hours	3818	18.1	(18.1, 20.6)	0.92	(0.83, 1.02)
Weather					
Sunny day	3081	19.0	(17.6, 20.4)	1.00	
Cloudy day	3151	19.8	(18.5, 21.2)	1.08	(0.95, 1.21)
Rainy/snowy day	1130	24.4	(22.0, 27.0)	1.01	(0.87, 1.19)
Foggy/windy/dusty day	249	20.9	(16.3, 26.4)	1.53	(1.13, 2.08) *
Installed camera at station					
Without	4713	20.9	(19.8, 22.1)	1.00	
With	2898	19.0	(17.6, 20.5)	0.89	(0.80, 0.99) *

*: *P* < 0.05;

IRR: incidence rate ratio.

### Traffic light signal violations

Of 5,612 observations at road intersections, 2.2% were coded as the driver ‘running traffic lights’ (95% CI: 1.9%- 2.7%) (Table 3). The incidence rate of running traffic light violations was lower on cloudy days compared to sunny days (adjusted IRR: 0.60).

**Table 3:** Incidence rate of violating traffic light signals by bus drivers (Changsha city, 2013).

Independent variables	Number of observations	Incidence rate		Adjusted IRR (95%*CI*)	
		%	(95% *CI*)		
Total	5612	2.2	(1.9, 2.7)		
Week					
Weekdays	2787	2.4	(1.9, 3.2)	1.00	
Weekend	2825	2.0	(1.6, 2.6)	0.92	(0.64, 1.33)
Time period					
Off-peak hours	2803	2.3	(1.8, 3.0)	1.00	
Peak hours	2809	2.1	(1.7, 2.7)	0.92	(0.65, 1.30)
Weather					
Sunny day	2186	2.7	(2.1, 3.4)	1.00	
Cloudy day	2521	1.6	(1.2, 2.2)	0.60	(0.39, 0.93) *
Rainy/snowy day	715	3.1	(2.0, 4.6)	1.07	(0.62, 1.82)
Foggy/windy/dusty day	190	2.6	(1.1, 6.0)	1.21	(0.44, 3.29)
Installed camera					
Without	1259	2.5	(1.7, 3.5)	1.00	
With	4353	2.2	(1.8, 2.6)	0.81	(0.54, 1.22)
Presence of traffic police					
Without	5440	2.2	(1.9, 2.6)	1.00	
With	172	2.9	(1.3, 6.6)	1.35	(0.55, 3.33)

*: *P* < 0.05;

IRR: incidence rate ratio.

### Distracted driving

Of 14,277 observations at road sections, 3.3% were coded as the driver driving while distracted (95% CI: 3.0%- 3.6%) (Table 4). The incidence rate of distracted driving was lower on cloudy days, rainy/snowy days, and foggy/windy/dusty days compared to sunny days, with adjusted IRRs of 0.54, 0.55, and 0.07, respectively.

**Table 4:** Incidence rate of distracted driving by bus drivers (Changsha city, 2013).

Independent variable	Number of observations	Incidence rate		Adjusted IRR (95%*CI*)	
		%	95% *CI*		
Total	14277	3.3	(3.0, 3.6)		
Week					
Weekdays	7126	3.1	(2.8, 3. 6)	1.00	
Weekend	7151	3.5	(3.1, 3.9)	1.12	(0.92, 1.35)
Time period					
Off-peak hours	7073	3.4	(3.0, 3.8)	1.00	
Peak hours	7204	3.3	(2.9, 3.7)	0.95	(0.79, 1.14)
Weather					
Sunny day	5806	4.2	(3.7, 4.8)	1.00	
Cloudy day	6069	2.4	(2.0, 2.8)	0.54	(0.43, 0.68) *
Rainy/snowy day	1929	4.2	(3.4, 5.1)	0.55	(0.42, 0.72) *
Foggy/windy/dusty day	473	0.4	(0.1, 1.5)	0.07	(0.02, 0.30) *
Installed camera					
Without	13153	3.3	(3.0, 3.6)	1.00	
With	1123	3.7	(2.7, 4.9)	0.86	(0.62, 1.19)

*: *P* < 0.05;

IRR: incidence rate ratio.

## Discussion

We reported the frequency of three types of traffic violations by bus drivers in urban China. Traffic violations were common. In particular, we found that drivers made illegal stops at bus stations over 20% of the time. Distracted driving and traffic light signal violations occurred less frequently, but did occur with some frequency. We found violation rates varied somewhat by weather and date.

The incidences of illegal stopping in Changsha city, China was much lower than that reported by by Mirza et al [16] in Karachi, Pakistan. In that study, incomplete stopping occurred 30% of the time, stopping outside of stations 46% of the time and stopping in the middle of the road 79% of the time. The differences may reflect differences in culture or road traffic management across countries, as well as differences in study time, as the Pakistani study was conducted in 1999. The fact that we discovered frequent violations at bus stations is concerning since stations present high risk sites for crashes and injuries [6]. The incidence of traffic light signal violations of bus drivers (2.2%) in Changsha city, China was much higher than similar reports by motor vehicle drivers (0.14%) in a different Changsha study [7]. The difference may be explained by the differing personal burden for traffic violation penalties among bus drivers versus private car drivers. The penalties to bus violations are generally stricter than to private car violations in China, but to maximize profit the bus companies require their drivers to complete routes in an expeditious manner, resulting in some motivation for violating traffic laws [17]. When drivers are charged with violations, typically the bus company will take charge of dealing with authorities to resolve and pay penalties, and therefore the actual penalties to bus drivers personally are far less than that to private car drivers. One strategy to improve bus safety may be to raise the governmental investment so as to change the profit-seeking behaviours of bus companies’ motives. Another method is to increase the penalty drivers personally absorb when they violate traffic laws.

We found that illegal stopping at bus stations was less common on weekends than weekdays, perhaps because the traffic volume is less and drivers can more easily abide by regulations. Interestingly, however, traveling during peak hours was not associated with bus driver violations. It may be that drivers are more vigilant during peak hours, and therefore despite the high traffic flow they do not violate laws with greater frequency.

We found that traffic cameras reduced illegal stopping at bus stations, suggesting camera monitoring might offer an effective strategy to reduce illegal driver behavior at stations. We did not find significant associations between use of traffic cameras and traffic light signal violations, however. This finding is consistent with a review by Aeron-Thomas and Hess [15] and may suggest methodological restrictions like not adjusting for regression to the mean. Large and controlled studies are needed [15].

As others have reported [12–14], we found the weather was associated with traffic violations by bus drivers. In particular, drivers must change their driving behaviors in bad weather [14]. We found high rates of illegal stopping on foggy/windy/dusty days, and decreased rates of violating traffic light signals and distracted driving on most bad weather days compared to sunny days. The conflicting results may be due to the individual judgment of bus drivers concerning potential risk in the different types of traffic violations in different types of bad weather.

Mirza et al [16] reported the surprising result that bus drivers had more high-risk behaviors in the presence of policemen in Pakistan and offered the explanation that traffic police may be more likely to be present at road intersections with high violation rates. We did not find any significant association between the presence of traffic police and running traffic light signals among bus drivers in Changsha city. This may be because traffic police are less common in China or because the burden of bus driver penalties is shared by bus companies in China. Further studies are needed to explore the role of traffic police on bus driver behaviour.

Our research had strengths and weaknesses. This is among the first study to observe violations among bus drivers in a large urban Chinese city. We used standard observational methods and collected data from a very large number of bus routes. Our research also suffered from limitations. First, the investigators’ vision was limited in some cases and on rare occasions (< 1% of observations), data were lost because observers could not clearly observe drivers in a crowded bus. Second, some drivers may change their behaviors when they realize they are being observed by investigators. Anecdotally, this seemed uncommon and most drivers were not aware of the ongoing research. Third, we did not assess the impact of individual difference factors concerning the bus drivers on their traffic law violations. Previous research suggests individual difference factors such as age, personality, and attributions are important factors that influence drivers’ traffic law violations [18–23] and future research might consider the effects of individual difference factors on bus drivers’ traffic law violations. Last, our research was conducted only in one urban Chinese city and it is unknown whether results would generalize to other cities or countries.

## Conclusion

Road traffic violations are common among bus drivers in Changsha city, China. The violations of bus drivers are associated with weather, type of day (weekday vs. weekend), and camera-based traffic management. High traffic violation rates of bus drivers call for attention from multiple stakeholders, including bus companies, traffic police, researchers, and policy-makers. Prevention strategies might include attention to enforcing traffic safety laws, instituting new laws targeting bus operation, and education or training of bus drivers concerning the risks involved in violating traffic safety principles.

Future research should consider other factors that may influence bus drivers’ traffic law violations, including individual factors (sex, age, personality, health status, and driving years), environmental factors, bus company management, and road traffic management.
